# Thundergust

**Published:** 1870-09

**Authors:** 


### THUNDERGUST.

Don't come down into the breakfast-room of a morning with a thundercloud over your pitiful phiz. What are you mad at? Has the servant forgotten to furnish you with a towel, or to leave your shoes at the door? Have you a note to pay in bank and not a dime to meet it? Have you the headache? Did the baby wake you from a sound sleep? Has the newsman failed to leave the morning paper; or has a bill been presented at your door bright and early, which you know very well ought to have been paid long ago to the patient baker, the needy dressmaker, or the begrimed mechanic? Maybe you cut your face while shaving, or stumped your toe against the cat, or broke your watch-spring in winding it up. Maybe you are ruffled because you have suddenly remembered that you failed to meet a business engagement yesterday; or took a short turn on a friend, or cheated somebody, and that it is about being found out, otherwise you would not have cared the snap of your finger about it; perhaps as you entered the door and scanned the breakfast table, you missed some favorite dish. But does one of all these occurrences justify you in clouding the whole household, in hurting the feelings of your children, in discouraging the servants, and outraging the guests at your table? The man or woman who comes to the family table with a scowl is a brute in nature, and only wants opportunity to bring out his littleness and his meanness. If, when you come to the breakfast-room, you are sad from discouragment, from fatigue, from illness, or from losses, you are excusable, and you have our sympathy; but to meet the family with a frown, and cast a gloom over the whole household, simply because everything has not gone exactly according to your sovereign pleasure, you, a poor pitiful worm of an hour ; why, it is so supremely ridiculous, that anything else than contempt for it is simply impossible.

He came into the breakfast-room one morning, and in a moment it was seen that a cloud was on his brow. There sat the ladylike wife waiting for him; the table fairly groaned, not with plated silver but the solid material. The cloth was white as the snow; the family were seated around in pleasant expectancy; everything was smoking hot, and not an article there but that even a pampered appetite could revel on. But the man's favorite dish was not there. Closer he came to the table, and with the inquiry, "Did you not know that I wanted a shad for breakfast?" he raised his foot and overturned the whole table on the floor.

"It was at the fire, being kept warm for you," replied his noble wife in her quiet, ladylike, and conquering way.

In an instant the haughty husband comprehended the situation; the next, he was on his knees exclaiming "Dear wife, you are nothing less than angel born." Not a great while after that he died. His will was opened; his wife was executor; he left her all he had, two millions of dollars.

